# Drainage and Irrigation in Urethrotomy

**Published:** 1900-03-24

**Authors:** 


### DRAINAGE AND IRRIGATION IN URETHROTOMY.

To cut through a stricture is one thing; to get, as a result, a pliable and useful urethra is quite another affair, and there is much reason for believing that this result is affected to a considerable extent by the care taken to drain and irrigate the wound. Mr. Reginald Harrison,[1] writing on the subject of external urethrotomy, says the entire character of the tissue by which a stricture is repaired after it has been divided, either in external or internal urethrotomy, may be importantly determined by the nature and duration of the drainage and irrigation that is employed, and he advises that after the stricture has been divided a full-sized gum elastic bladder drainage tube should be employed, for which after a few days a flexible rubber one may be substituted. The kind of tube to be used is one made in gum elastic in different lengths and sizes, so as to fit the wound and the deep urethra with as much accuracy as a tracheotomy tube fits a windpipe. From the day of its insertion it commences to mould the repairing urethra to the dimensions required. It is readily inserted, and can be removed for purposes of irrigation and cleanliness; and when any risk of bleeding or oozing, for which side-packing might be required, is over its place is taken by a soft rubber tube with depressed openings of similar dimensions. Thus cicatrisation proceeds under free irrigation up to the time of the final removal of the tube.

[1] Lancet, Mar. 17.

